# Aspirin Interacts with Cholesterol-Containing Membranes in a pH-Dependent Manner

**DOI:** 10.1021/acs.langmuir.3c02242

**Published:** 2023-11-08

**Authors:** Michael Krmic, Escarlin Perez, Patrick Scollan, Katherine Ivanchenko, Alondra Gamez Hernandez, Joseph Giancaspro, Juan Rosario, Jasmin Ceja-Vega, Jamie Gudyka, Riley Porteus, Sunghee Lee

**Affiliations:** Department of Chemistry and Biochemistry, Iona University, 715 North Avenue, New Rochelle, New York 10801, United States

## Abstract

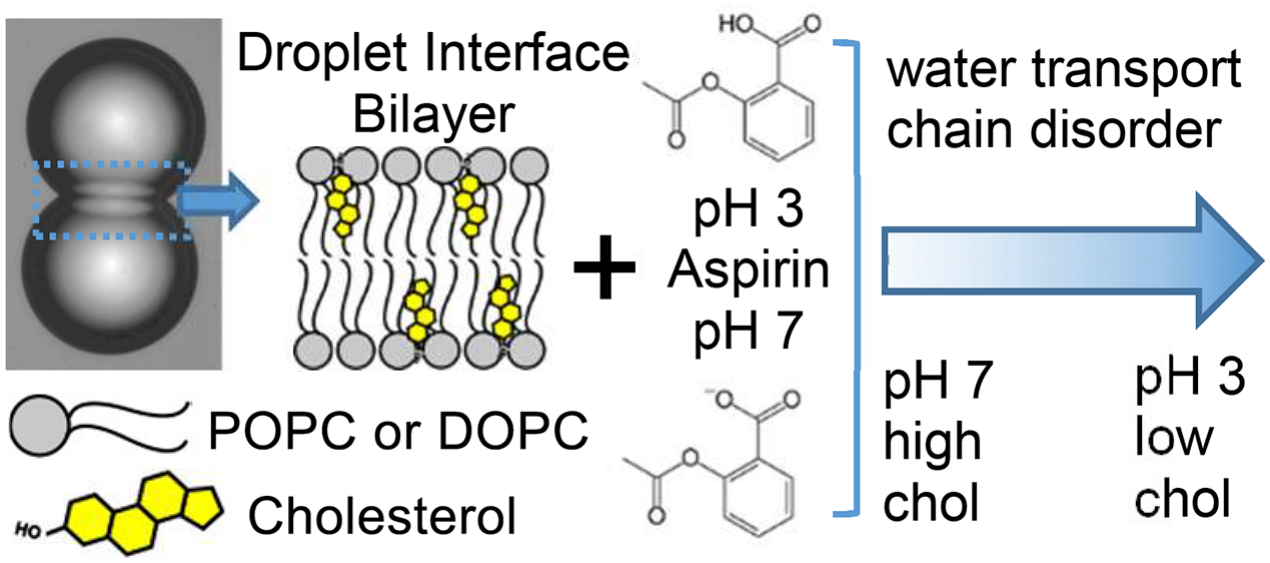

Aspirin has been used for broad therapeutic treatment, including secondary prevention of cardiovascular disease associated with increased cholesterol levels. Aspirin and other nonsteroidal anti-inflammatory drugs have been shown to interact with lipid membranes and change their biophysical properties. In this study, mixed lipid model bilayers made from 1-palmitoyl-2-oleoyl-*sn*-glycero-3-phosphatidylcholine (POPC) or 1,2-dioleoyl-*sn*-glycero-3-phosphatidylcholine (DOPC) comprising varying concentrations of cholesterol (10:1, 4:1, and 1:1 mole ratio of lipid:chol), prepared by the droplet interface bilayer method, were used to examine the effects of aspirin at various pH on transbilayer water permeability. The presence of aspirin increases the water permeability of POPC bilayers in a concentration-dependent manner, with a greater magnitude of increase at pH 3 compared to pH 7. In the presence of cholesterol, aspirin is similarly shown to increase water permeability; however, the extent of the increase depends on both the concentration of cholesterol and the pH, with the least pronounced enhancement in water permeability at high cholesterol levels at pH 7. A fusion of data from differential scanning calorimetry, confocal Raman microspectrophotometry, and interfacial tensiometric measurements demonstrates that aspirin can promote significant thermal, structural, and interfacial property perturbations in the mixed-lipid POPC or DOPC membranes containing cholesterol, indicating a disordering effect on the lipid membranes. Our findings suggest that aspirin fluidizes phosphocholine membranes in both cholesterol-free and cholesterol-enriched states and that the overall effect is greater when aspirin is in a neutral state. These results confer a deeper comprehension of the divergent effects of aspirin on biological membranes having heterogeneous compositions, under varying physiological pH and different cholesterol compositions, with implications for a better understanding of the gastrointestinal toxicity induced by the long term use of this important nonsteroidal anti-inflammatory molecule.

## Introduction

Acetylsalicylic acid (ASA), commonly referred to as aspirin, is a long-used drug commonly indicated for broad therapeutic treatment including alleviating pain, fever, and inflammation.^1,2^ In particular, a regimen of aspirin at low-doses had been widely employed for its putative efficacy in secondary prevention of cardiovascular disease (CVD)^3^ and cholesterol related diseases in which plasma cholesterol levels have been associated with the risk of CVD.^4^ Diseases of the cardiovascular system consistently rank among the top ten leading causes of death in the United States, responsible for more than 1 in 4 deaths, according to 2017 mortality statistics.^5^ The beneficial effect of aspirin for the secondary prevention of CVD has been established;^6^ however, the efficacy of aspirin for CVD primary prevention has proved to be controversial^7^ and led to a new Recommendation Statement from the U.S. Preventive Services Task Force.^8^

Aspirin is considered to be unique among nonsteroidal anti-inflammatory drugs (NSAIDs), as it irreversibly blocks activity of cyclooxygenase (COX), a monotopic membrane protein that mediates the inflammatory process.^9^ These properties make aspirin a potential cardiovascular-protective agent, having long duration of its action (7 to 10 days after drug discontinuation) as well as having accompanying risk.^10^ In comparison, most non-aspirin NSAIDs reversibly inhibit COX enzyme.^10^ The long term use of aspirin even in low-dose has been reported to have toxicity, including gastric ulceration and other gastro-intestinal (GI) complications from its induced side effects.^11^

A growing body of evidence implicates a critical role for interactions of NSAIDs with biological membranes, and hence, there is a need for better understanding.^12^ Changes to the collective structural and physical properties of lipid membranes would have functional consequences for membrane-bound proteins and consequently significant implications on the proper physiological functions of cells.^13−15^ There have been reports suggesting that a direct interaction of aspirin with the phospholipid membrane of cells of the gastric mucosa is responsible for local cytotoxic effects..^16−18^ Previous experimental and computational work has shown that the interaction of aspirin with the cellular membrane can modulate various biophysical properties of the phospholipid bilayer, such as its compressibility, area per molecule, thickness, lipid packing, and cooperativity of hydrocarbon chains.^19−26^ For example, analytical techniques including SANS (small-angle neutron scattering) and neutron spin echo give evidence for a plasticizing effect of aspirin on 1,2-dimyristoyl-sn-glycero-3-phosphocholine (DMPC) model membranes in both gel and fluidic phase, manifesting an effect on the fluidity and flexibility of membranes.^24^ Using neutron spin echo, aspirin has been shown to enhance the microscopic lipid dynamics in DMPC membrane to soften the bilayer.^23^ In model studies of DMPC with cholesterol, ASA has been shown to dissolve excess cholesterol patches.^20^ Using neutron diffraction, it was reported that ASA is able to locally disrupt organization of the liquid-ordered membrane formed by 1,2-dipalmitoyl-*sn*-glycero-3-phosphatidylcholine (DPPC) with cholesterol, and inhibit cholesterol-raft formation.^21^ This stands in contrast to a suite of studies showing that intercalation of amphipathic NSAID molecules into raft-forming membranes can in fact promote further phase separation and stabilization of coexisting L_o_/L_d_ domains.^27,28^

The protective hydrophobic barrier of the cell, which separates the cellular interior from its surrounding environment, is the lipid bilayer membrane. Such protective ability is especially important in the context of the gastrointestinal tract, where an extracellular mono/multilayer of phospholipids on the surface of the mucus gel layer attenuates the damaging action of NSAIDs.^18^ In general, the cell membrane is highly complex and diverse, consisting of a wide variety of different lipid constituents that take an asymmetric form with respect to the lipid components of respective leaflets.^29^ The main kind of lipid component found in plasma membranes of cells of the human gastrointestinal (GI) tract is phosphatidylcholine (PC) with unsaturated hydrocarbon chains (such as 1-palmitoyl-2-oleoyl-*sn*-glycero-3-phosphocholine (POPC, 16:0/18:1 PC)), as well as cholesterol.^30^ Cholesterol plays a vital role in the functional, structural, and dynamic properties of membranes, including regulation of transmembrane proteins and modulation of membrane fluidity.^31^ As a key component of lipid rafts, cholesterol serves to keep the raft assembly together, and a disturbance in concentration has been linked to a variety of diseases of lipid metabolism.^32^ In view of a close association of ASA usage with many cholesterol-related disease treatments, it is essential to obtain an enhanced understanding of the interaction of ASA with membranes under varying conditions of cholesterol content, in order to more appropriately evaluate the physiological activity and cytotoxicity of ASA.

In general, >60% of the drugs used for humans are ionizable, and the extent to which they ionize depends on environmental pH and intrinsic pK_a_, affecting their bioactivity. Especially along the gastrointestinal tract, ionizable drugs will encounter varying pH levels.^33^ The physical-chemical properties of drugs at different pH values will affect their hydrophilic/lipophilic balance and thus membrane permeability.^34^ This speaks to a broad importance of pH-dependent membrane interactions, which extends beyond pharmaceuticals to many other membrane-bound substances such as protein toxins and peptides.^35^

**Figure 1:**
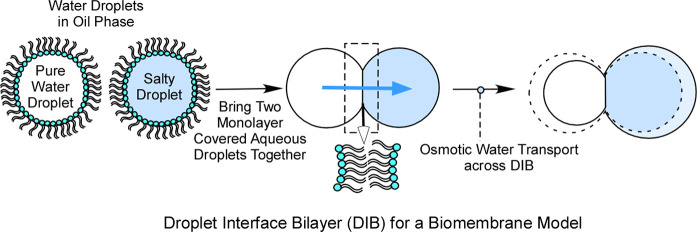
Schematic of aqueous microdroplets surrounded by self-assembled structures, for use as a biomembrane model, provides a platform to study DIB-based osmotic water permeability measurement

In this paper, we have investigated the influence of ionizable ASA molecules on the physical properties of POPC and DOPC model membranes having varying concentrations of cholesterol as a function of ASA concentration under acidic (pH 3) and neutral (pH 7) conditions, i.e., below and above the pK_a_ of ASA, respectively. ASA has a pH-dependent charge state (pK_a_ ≈ 3.50), with protonated uncharged state at acidic pH (pH < pK_a_), while it is in charged state at neutral pH.^19,36^ These differing charge states of ASA may result in different lipid membrane interactions depending on the surrounding physiological pH values in the stomach and the large intestine. To assess changes in the bilayer physical properties, the parameter of passive water permeability was used as a metric to gauge perturbations in membrane organization owing to ASA interactions. In general, the process of water transport is a function of the physical state of the lipid bilayer and its structure,^37^ and water permeability itself has significance for cellular homeostasis and physiology. In our earlier studies, we have established a reliable method for quantifying water transport through lipid bilayers using the droplet interface bilayer (DIB) as membrane model.^38,39^ One can form a DIB, by contact of two aqueous droplets when positioned in a surrounding immiscible medium and decorated with a lipid monolayer, to provide a bilayer region at the point of mutual contact (Figure 1). This molecular structure in this region is essentially that of the double leaflet lipid bilayer structure in cellular membranes.^40,41^ Our previous findings demonstrate the versatility of the DIB in providing flexible levers for probing structural effects in self-assembled lipidic amphiphiles,^42−45^ including exploration of the effects of exogenous molecules on membrane properties.^46−48^ Additional methods were employed for investigating the interplay between the model membrane and ASA in its differing charge states, including differential scanning calorimetry (DSC) of multilamellar vesicles (MLVs), confocal Raman microspectroscopy of supported bilayers, and interfacial tensiometry. Our data show that the interaction of ASA with model cellular membranes (POPC and DOPC) depends on the composition (concentration of cholesterol), drug concentration, and charge state of drug. Specifically, we show that ASA appears to counteract the condensing effect of cholesterol in lipid bilayer assemblies and that the extent of this counteracting effect depends on cholesterol concentration and the charge state of ASA, where the effect is greater for uncharged ASA.

## Materials and Experimental Details

### Preparation of Materials and Samples

Structures of the principal compounds employed in the present study are shown in Table 1. PC lipids were purchased from Avanti Polar Lipids, Inc. (Alabaster, AL) at a purity level of 99+% and used in form received. POPC and DOPC were each provided as a solution in chloroform. Squalene (2,6,10,15,19,23-hexamethyl-2,6,10,14,18,22-tetracosahexaene; C_30_H_50_; SqE), cholesterol (chol), and ASA were procured in their highest available purity level from Sigma-Aldrich and used without additional purification. All samples of PC lipids and chol were freshly prepared immediately before use in experiments or stored at −20 °C until use. In order to mitigate photo-oxidation of unsaturated lipids (DOPC and POPC), the relevant sample solutions are prepared in an amber bottle or wrapped with aluminum foil. To avoid decomposition, SqE was kept at a temperature in the range of 2 °C – 8 °C. Preliminary to the preparation any lipid-containing oil solution, a CHCl_3_ solution of lipid (or lipid and chol) is placed in a vial and the solvent evaporated under inert gas flow to produce a dried lipid thin film, which is thereafter dried overnight under vacuum for complete solvent removal. Analogously, for preparation of oil solutions including ASA, the ASA is codissolved with lipid in chloroform, followed by solvent evaporation to generate a dried film of defined ASA/lipid mole ratio, which is thereafter resuspended in SqE. The total lipid concentration (in the oil phase) used for all water permeability experiments was 5 mg/mL of SqE. When mixtures of PC lipid and chol were used, three different ratios were employed, namely, 10:1, 4:1 and 1:1 mole ratio of PC lipid:chol. For sample preparations used in DSC experiments, dried ASA/lipid films described above are subsequently rehydrated with a phosphate buffered solution (10 mM) at pH 3 or pH 7 made with deionized water (18.2 MΩ·cm, Direct Q-3 Millipore water purification system). The total lipid concentration used for DSC was ca. 16 mg/mL. To obtain a suspension of MLVs, the rehydrated liposome suspension was vigorously vortexed for about 5 min and then subsequently exposed to to bath sonication for about 30 min. To obtain samples suitable for confocal Raman microspectroscopy, an MLV suspension prepared as above was treated with seven freeze-thaw cycles (liquid N_2_), and subsequently deposited on a glass substrate. A vapor pressure osmometer (VAPRO model 5600) was employed to measure the osmolality (mOsm/kg) of aqueous solutions used for water permeability, immediately after fresh preparation and just prior to the experiment.

**Table 1:**
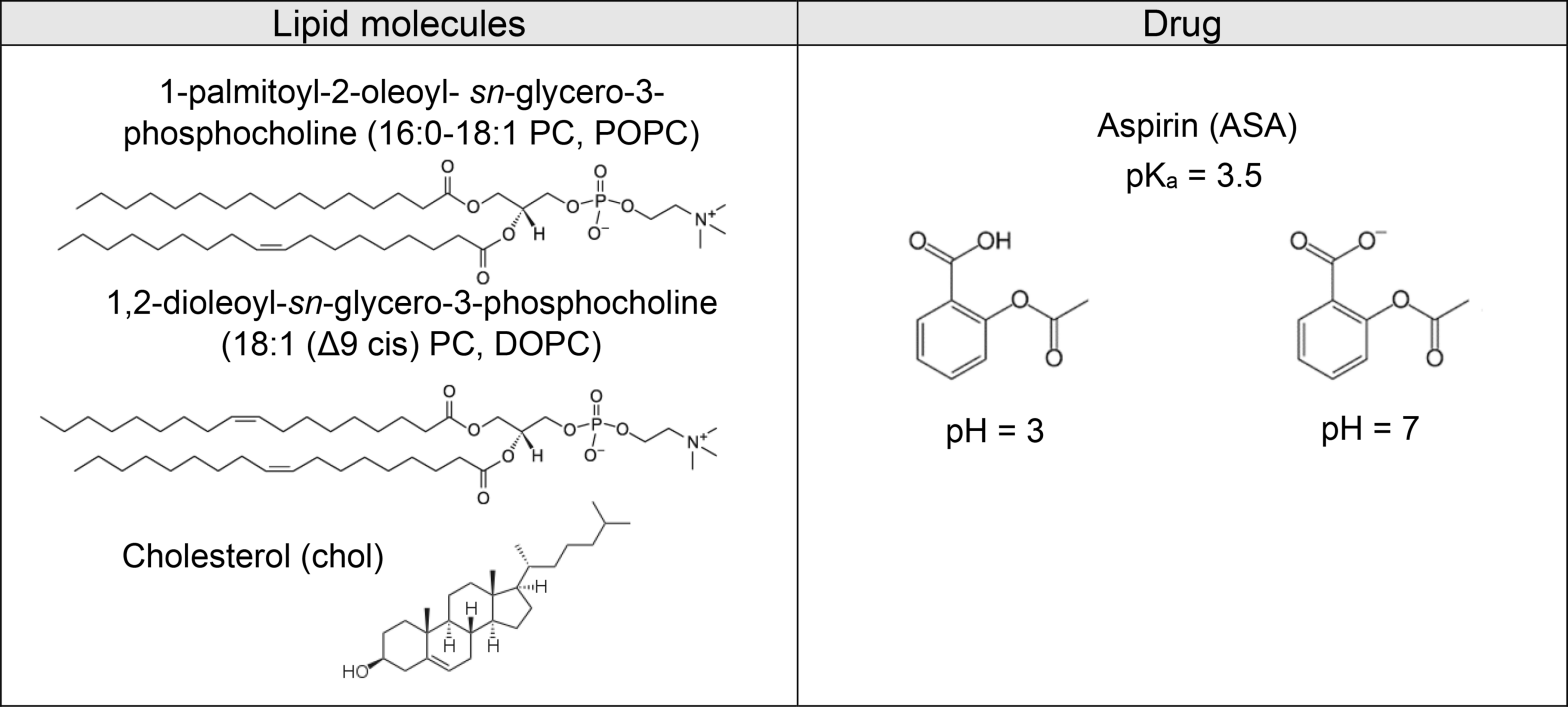
Structures of POPC, DOPC, Cholesterol, and Aspirin Molecules

### Experimental Details

#### Water Permeability Measurement

The experimental practices and procedures for extraction of water permeability parameters using the DIB method have been set forth in prior publications, and a similar setup has been used for this experiment.^42^ A setup consists of a micropipet manipulation station built on an inverted microscope with a camera directly attached to the microscope for real time recording of the microdroplets and their size changes. In brief, a pair of osmotically unbalanced aqueous droplets is created in an immiscible solvent (SqE) in which are dissolved ASA and lipid mixtures, at a given mole ratio. When an osmotic pressure imbalance exists between two adhering aqueous droplets in a DIB, water transport occurs through the droplet bilayer, leading to a measurable change in droplet diameter (Figure 1). The squalene solution is held between glass strips, and the droplets are introduced via the micropipets. The reader is referred to our previous papers for more details.^42^ A constant temperature of 30 °C was set for all water permeability experiments and ensured by a custom-built temperature-controlled microchamber that was thermostated via a circulating water bath. The data points presented herein represent the mean of individual permeability runs (*n* > 30). To measure the dimensions of individual droplets and contact area between adherent droplets, the recorded videos were postanalyzed using custom-built image analysis software. The detailed method of water permeability calculation is provided in Supporting Information.

#### Differential Scanning Calorimetry (DSC) Measurements

DSC measurements were performed using a TA Q2000 DSC instrument. The samples were aqueous dispersions of MLVs of DOPC (or DOPC with chol at varying mole ratios) containing defined concentrations of ASA at the two chosen pH levels. The TA Universal Analysis software was used to ascertain the main phase transition temperature (*T*_m_), the apex temperature for the endothermic transition peak, and the enthalpy of phase transition (Δ*H*), integrated area under the heat capacity curve. DSC runs employed aliquots of about 15 μL of the MLV dispersions prepared as described in the sample preparation section. These were hermetically sealed and subjected to heating/cooling under high purity nitrogen with a flow rate of 50 ml/min, at rates of 5 °C/min from −40 °C to 0 °C. All experiments are repeated with three independently prepared samples, and each sample was cycled three times. Reproducible results were obtained, and no hysteresis was observed.

#### Confocal Raman Microspectroscopic Measurements

The Raman spectra of supported lipid bilayers including varying concentrations of ASA molecules were obtained by employing confocal Horiba XploRA INV (Nikon Eclipse Ti-U) instrumentation, having as a light source an internal air-cooled solid-state laser at 532 nm, with a cooled CCD detector. Aliquots of paucilamellar vesicle suspension (10 to 20 μL), obtained immediately after being subjected to a freeze-thaw process (as per the sample preparation section), were allowed to spread onto glass coverslips (#1.5). A solid supported lipid bilayer (SSLB) was obtained upon removal of residual aqueous solvent using a heating plate at about 30 °C in a closed chamber. At least three independent samples are prepared, and multiple scans (3–4 regions) in a given sample were averaged with 20 accumulations using a 40x microscope objective (N.A.0.60) and a grating of 1200 lines per millimeter. All spectroscopic experiments were performed at ambient temperature.

#### Interfacial Tension and Contact Angle Measurements

The interfacial tension at the oil-water interface was measured using a ramé-hart Advanced Goniometer/Tensiometer (Model 590), with postanalysis of obtained images using the software DROPImage. An oil droplet containing DOPC (or DOPC with chol), dissolved in SqE, containing a given mole ratio of ASA, is created in the aqueous phase. For the interdroplet contact angles (θ), two juxtaposed iso-osmotic droplets in the surrounding oil phase are made to contact with each other. The contact angle can be derived from the microscopic video images of the two adherent droplets, by considering the geometry of the contacting spheres (evaluated using equation 1) based on geometrical parameters shown in Supporting Information (Figure S2),
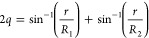
1where *R*_1_ and *R*_2_ are the respective radii of the two droplets and *r* is the radius of the inter-droplet contact zone. The mean values from 10 or more measurements were reported for these parameters.

## Results and Discussion

### Water Permeability

**Figure 2:**
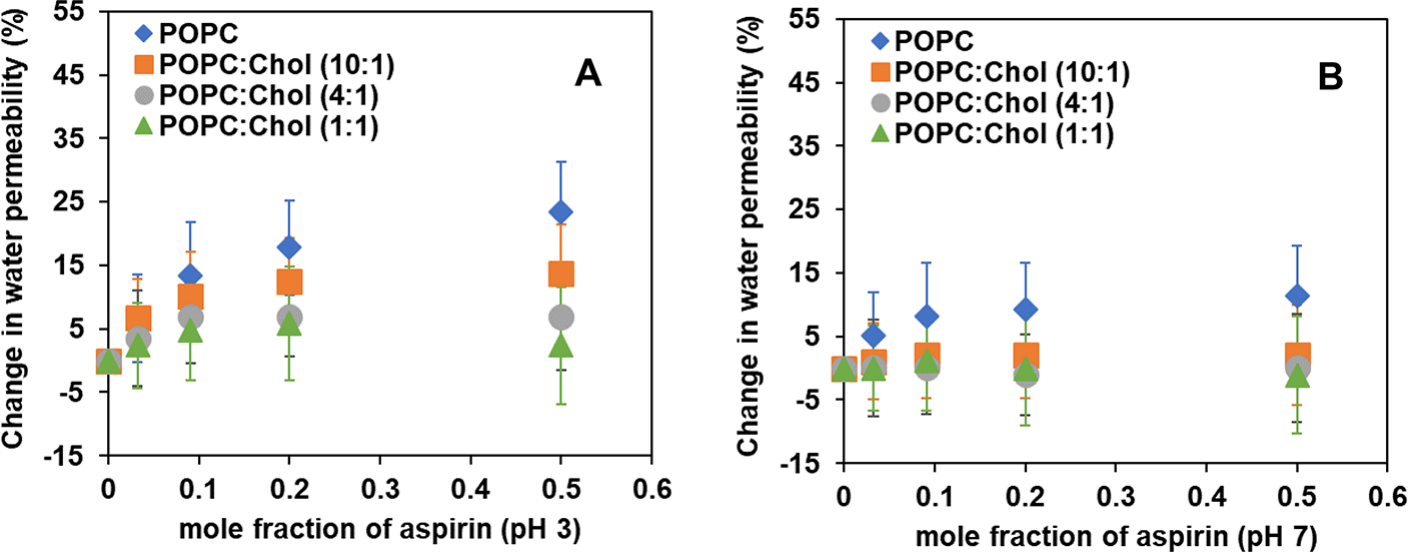
The relative percentage change (%) in osmotic water permeability (*P_f_* /*P*_*f*_^*o*^, where *P*_*f*_^*o*^ represents the osmotic water permeability in the absence of ASA) of POPC and mixed bilayer formed from POPC:chol at 30 °C with varying mole fraction of ASA, at (A) pH 3 and (B) pH 7.

We report the changes in osmotic water permeability across lipid bilayers of various compositions at two different pH values, as a percentage change relative to the absence of ASA from that lipid bilayer: *P_f_* /*P*_*f*_^*o*^, where *P*_*f*_^*o*^ represents the osmotic water permeability of a given lipid bilayer in the absence of ASA at 30 °C, and *P_f_* is the relevant parameter in presence of ASA. Figure 2A shows the parameters obtained for systems with ASA in uncharged state (pH 3), and Figure 2B illustrates the values for charged ASA (pH 7). The corresponding numerical values for coefficients of osmotic water permeability (*P_f_*) across these same lipid bilayers at both pH 3 and 7, respectively, are shown in Table S1 of the Supporting Information. As seen in Figure 2 and Table S1, the water transport parameter *P_f_* for phosphocholine bilayers at 30 °C (in the absence of cholesterol, blue diamond) increases with increasing concentration of ASA at both pH 3 and pH 7. This water permeability increase occurs to a greater extent at pH 3 (Figure 2A) than at pH 7 (Figure 2B). As ASA concentration in the POPC bilayer increases from 1:0 (no ASA) to 1:1 mol ratio of POPC:ASA (χ_ASA_ = 0.5, the highest concentration of ASA we studied), *P_f_* of POPC increases approximately 24% (from 71 to 88 μm/s) at pH 3, i.e., presence of uncharged aspirin. When ASA is charged (pH 7), there is a 10% increase in water permeability at the same mole ratio (from 77 μm/s to 85 μm/s). Lesser values of ASA content (e.g., χ_ASA_ = 0.1 or 0.2) show lower permeability increases.

For the case of cholesterol-containing bilayers (POPC:chol at 10:1, 4:1 and 1:1 mol ratio), the increases in osmotic water permeability due to the presence of ASA are significantly attenuated relative to the POPC-only bilayers. At a low level of cholesterol inclusion (10:1 POPC:chol mole ratio, orange square in Figure 2), the addition of ASA (total lipid:ASA at 1:1) increased the osmotic water permeability by 12% (from 68 μm/s to 77 μm/s) at pH 3. But, only a 3% increase in water permeability at the like chol concentration was seen for pH 7 (from 73 to 75 μm/s at total lipid:ASA at 1:1). A similar trend for water permeability is observed for higher concentrations of chol, *viz.*, 4:1 (gray circle) and 1:1 (green triangle) mole ratio of POPC:chol at pH 3; however, the extent of change in water permeability is further lessened with increased concentration of chol in the POPC bilayer (8% increase, from 65 μm/s for ASA-free to 70 μm/s for 4:1 mole ratio of POPC:chol and high [ASA]; 2% increase, from 62 μm/s for ASA-free to 63 μm/s for 1:1 mole ratio of POPC:chol and high [ASA]). At pH 7 and at higher concentrations of cholesterol in POPC (4:1 and 1:1 POPC:chol mole ratio), there seems to be no significant differences in water permeability. Overall, the uncharged ASA increases water permeability in both the absence and presence of cholesterol at all ASA concentrations studied. However, at pH 7, such an increase in water permeability is seen only in the absence of chol or at low chol concentration (10:1 POPC:chol), and no such effect is seen at the relatively higher concentrations of cholesterol (4:1 and 1:1 POPC:chol). Markedly similar qualitative trends are found when DOPC instead of POPC is used as a membrane component (results are shown in Supporting Information, Table S2 and Figure S3).

The evident increase in water permeability coefficient for POPC membrane bilayers containing steadily increasing amounts of ASA is indicative of the nature and extent of the interactions of ASA with the membrane. It has generally been considered that water permeability rates are dependent upon the physical state of the lipid bilayer aggregate, such as its bilayer thickness and fluidity, and area per lipid.^49,50^ Previous reports suggest that thickness changes and modifications of bilayer fluidity will affect the water permeability.^51,52^ There is a correlation between the fluidity of bilayers and the lipid packing density, and water permeability is strongly anticipated to depend on a perturbation of lipid packing or the creation of porous voids in the bilayer region. The present findings, in which an increased ASA content is correlated with an increase in water permeability, are in general qualitative agreement with several previous observations in the literature regarding the impact of ASA on membrane fluidizing properties. For example, incorporation of ASA has been shown to fluidify DMPC model membranes, thinning this lipid bilayer, and reducing bending rigidity and compressibility modulus.^23,24^ Collectively, these findings point to the major influence that ASA can have upon the lipid bilayer, resulting in various structural perturbations that would support our findings of an increase in water permeability. In addition, the observed pH dependency for water permeability in the presence of ASA, where an increased value of water permeability is exhibited at pH 3 compared pH 7, is consistent with atomistic MD simulations demonstrating different modes of molecular interactions of ASA with the lipid bilayer: ASA in uncharged form (pH < pK_a_) partitions into the acyl chain region of DPPC, and perturbs the bilayer structure, but, in charged form (pH > pK_a_) is positioned towards the hydrophilic headgroup interface.^19^ The reported partition coefficients of ASA, log(*K*_OW_), at pH 2.0 and pH 7.4 are 1.13 and −1.20, respectively.^36^

In the absence of ASA, we found that increasing concentration of chol in POPC bilayers lead to reduction in water permeability of POPC, from 71 to 62 μm/s at pH 3, and from 77 to 67 μm/s at pH 7, for 1:1 mol ratio of POPC:chol (Table S1). Such reduction in water permeability by chol is qualitatively consistent with the literature, where the effect of chol on POPC has been shown to impose order upon a POPC bilayer while reducing its fluidity and dynamics.^31^ At concentrations between 5 and 25 mol% chol the bilayer becomes rigid, as shown by small-angle X-ray scattering experiments.^53^ It has also been reported that cholesterol and POPC bilayer at a 1:1 mol ratio exist in an ordered lattice arrangement. The condensation effect of chol on POPC and DOPC bilayer is reported to be similar in the range of mole fraction up to 0.5.^54^ Similarly, a reduction of permeability of small molecules across large unilamellar vesicle membranes composed of POPC is seen in the presence of increasing concentrations of chol, as studied by stopped-flow fluorimetry which monitors concentration-dependent or pH-sensitive quenching of encapsulated carboxyfluorescein.^55^

A summary of the systematic studies reported here reveals a greater extent of increase in water permeability when ASA is in the neutral charge state (Figure 2A) for all chol concentrations (from 10:1, 4:1 to 1:1 POPC:chol mole ratios). However, this effect is relatively muted at pH 7 with a 10:1 POPC:chol mole ratio, and there is almost no change in water permeability at higher concentrations of cholesterol (4:1 and 1:1 POPC:chol mole ratios, Figure 2B). That the presence of ASA leads to an increase in water permeability for a lipid-chol mixed bilayer illustrates its ability to fluidize or disorder a membrane which is otherwise in a relatively condensed liquid-ordered state. Prior studies (using inelastic neutron scattering combined with MD simulations) of DMPC containing chol (30 mol%) showed that ASA (10 mol%) interacts with the cholesterol-rich liquid-ordered phase (raft-like domains) leading to local fluidization changes with increasing area per lipid, suppressing cholesterol's ordering effect via direct binding of ASA molecules to chol.^22^ Langmuir–Blodgett experiments have shown that ASA (3 mM in the subphase) increases the area per lipid and decreases compressibility of DPPC membranes containing cholesterol (32.5 mol% of chol), indicating an increase in membrane fluidity.^21^ An increasing amount of ASA in DMPC was reported to increase the fluidity of the bilayers with a high concentrations of chol (40 mol%), by using X-ray diffraction study.^20^ These reports are qualitatively consistent with our findings, where increased water permeability results from ASA inclusion in the chol containing POPC bilayers. While direct quantitative comparison cannot be made for data obtained by differing experimental techniques and lipids used (e.g., literature data is for saturated PCs whereas our study used unsaturated PCs), our water permeability data provides additional evidence for ASA having a capability to counteract the condensing effect of chol in lipid bilayers.

### Thermotropic Property

**Figure 3:**
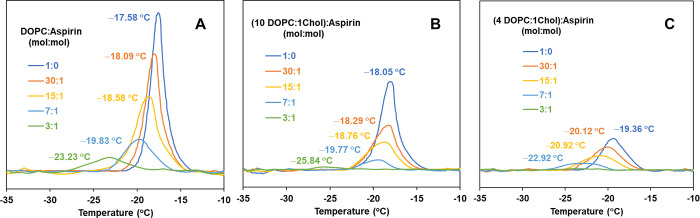
Endothermic calorimetric thermograms of DOPC and DOPC:chol MLVs containing different concentrations of ASA at pH 3: (A) DOPC, (B) DOPC:chol at 10:1 mol, and (C) DOPC:Chol at 4:1 mol. The same scale of the y-axis is used for relative comparison of Δ*H* for different composition of MLVs.

The endothermic DSC thermograms for pure DOPC and mixed DOPC:chol MLVs in the presence of different concentrations of ASA at pH 3 are shown in Figure 3. The corresponding thermodynamic data are tabulated in Table S3 of the Supporting Information, namely, *T*_m_ and Δ*H* at pH 3 as well as pH 7. Note that for DSC, we elected to employ DOPC as a base lipid instead of POPC, since the phase transition behavior of POPC MLVs (*T*_m_ = ∼2 °C) was found to be ill-defined, likely owing to interference from ice formation. On the contrary, DOPC MLVs under our experimental conditions provide well-defined thermograms. The detailed parameters used to obtain these DSC thermogram are given in the experimental section. The thermogram of pure DOPC MLVs (i.e., no aspirin, Figure 3A, most intense peak) shows an endothermic transition with pronounced definition, attributable to the transition of the lamellar gel phase L_β_ to the lamellar liquid-crystalline state L_α_ at the transition temperature (*T*_m_) of −17.58 °C, associated with an enthalpy of 9.59 kcal/mol, consonant with the literature.^56^ This low-temperature *T*_m_ evidence that the DOPC bilayer is in a disordered fluidic state, generally associated with the presence of unsaturated acyl chains. Figure 3A also shows that the main phase transition of the DOPC is prominently affected by inclusion of ASA in a concentration-dependent manner, shifting toward lower *T*_m_, and evincing an overall broadening with significant reduction in Δ*H*. For these pure DOPC membranes (i.e., in the absence of cholesterol), when ASA is included at 30:1 mole ratio of DOPC:aspirin, *T*_m_ is decreased by 0.5 °C (from −17.58 °C to −18.09 °C) with a reduction of about 20% in Δ*H* compared to DOPC alone (from 9.59 kcal/mol to 7.67 kcal/mol). These values are further decreased with increasing mole ratios of ASA, and at the highest concentrations of ASA used in DSC, 3:1 mole ratio of DOPC:ASA, the peak is significantly suppressed, as evidenced by a reduction of its enthalpy from 9.59 kcal/mol (DOPC) to 1.33 kcal/mol, and the main phase transition (*T*_m_) is shifted to a lower temperature by 5.6 °C (from −17.58 °C to −23.23 °C). These changes in phase behavior provide evidence for increasing fluidity in DOPC bilayers in the presence of ASA. Similar qualitative trends were observed for DOPC MLVs with aspirin at pH 7 (Supporting Information, Table S3), with a slightly lesser extent of perturbation compared to the thermogram at pH 3.

The addition of chol changed the thermotropic properties of pure DOPC, i.e., with no ASA present (Figure 3B and 3C, blue trace and Table S4). The main phase transition (*T*_m_) is shifted to a lower temperature (from −17.58 °C for pure DOPC to −18.05 °C for DOPC:chol at 10:1 mole ratio and to −19.36 °C for DOPC:chol at 4:1 mole ratio). The peak is suppressed, from 9.59 kcal/mol for pure DOPC to 6.14 kcal/mol for DOPC:chol at 10:1 mole ratio, and to 3.18 for DOPC:chol at 4:1 mole ratio. At an excessively high concentration of chol (1:1 DOPC:chol mole ratio), no apparent peak and no measurable transition enthalpy was observed. These results are consistent with previous reports on the thermotropic properties of DOPC MLVs as a function of chol content.^57,58^ To study the effect of ASA at pH 3, then, DOPC:chol mixtures at a mole ratio of 10:1 and 4:1 were further investigated. As seen in Figure 3B,C, the thermograms produced by progressive inclusion of ASA to chol-containing bilayers evidenced an overall set of changes which have qualitative similarities to pure DOPC in the presence of aspirin (Figure 3A). That is, *T*_m_ shifts to lower temperatures, and the peak is suppressed. Similar qualitative trends were observed for DOPC:chol MLVs with aspirin at pH 7 (Supporting Information, Table S4).

The differences observed in the bilayer thermotropic properties (both *T*_m_ and Δ*H*) of the phase transitions are taken to indicate that ASA can interact with both chol-free and chol-enriched DOPC MLVs to induce changes in the conformation of lipid acyl chains and/or the lipid headgroup region, likely influencing the lipid packing as well. These findings are qualitatively consistent with previous DSC studies, albeit for saturated PCs. For example, it has been reported that addition of ASA results in a decrease in the *T*_m_ and broadening of the main transition peak (and elimination of the pre-transition) for DPPC MLVs, interpreted as evidence for a reduction in cooperativity of the main phase transitions as a result of a more fluid structure.^21^ Similar results were reported for DMPC MLVs, where the presence of 6.5 wt% ASA has also been shown to shift the main phase transition peak toward lower temperature with significant broadening.^23^ In sum, ASA in both neutral and charged state can modulate the lipid arrangement by interaction at the acyl chain region and/or lipid headgroup region, changing both phase transition temperature (*T*_m_) and enthalpy (Δ*H*) of the phase transition from lamellar gel phase to the lamellar liquid-crystalline state. Note that MLVs were used for DSC studies due to the advantage of greater amount of lipidic sample which exhibit more cooperativity in their lipid phase transitions compared to unilamellar vesicles.^59^ However, it is recognized that surfactant-drugs (such as the aspirin that is the focus of this study) would partition in various regions of a multilayer membrane,^60^ and so drug concentration may not be the same in outer lipid layers as in inner ones; and water is largely absent from the midregion of a multilayer.

### Structural Properties by Vibrational Spectroscopy

**Figure 4:**
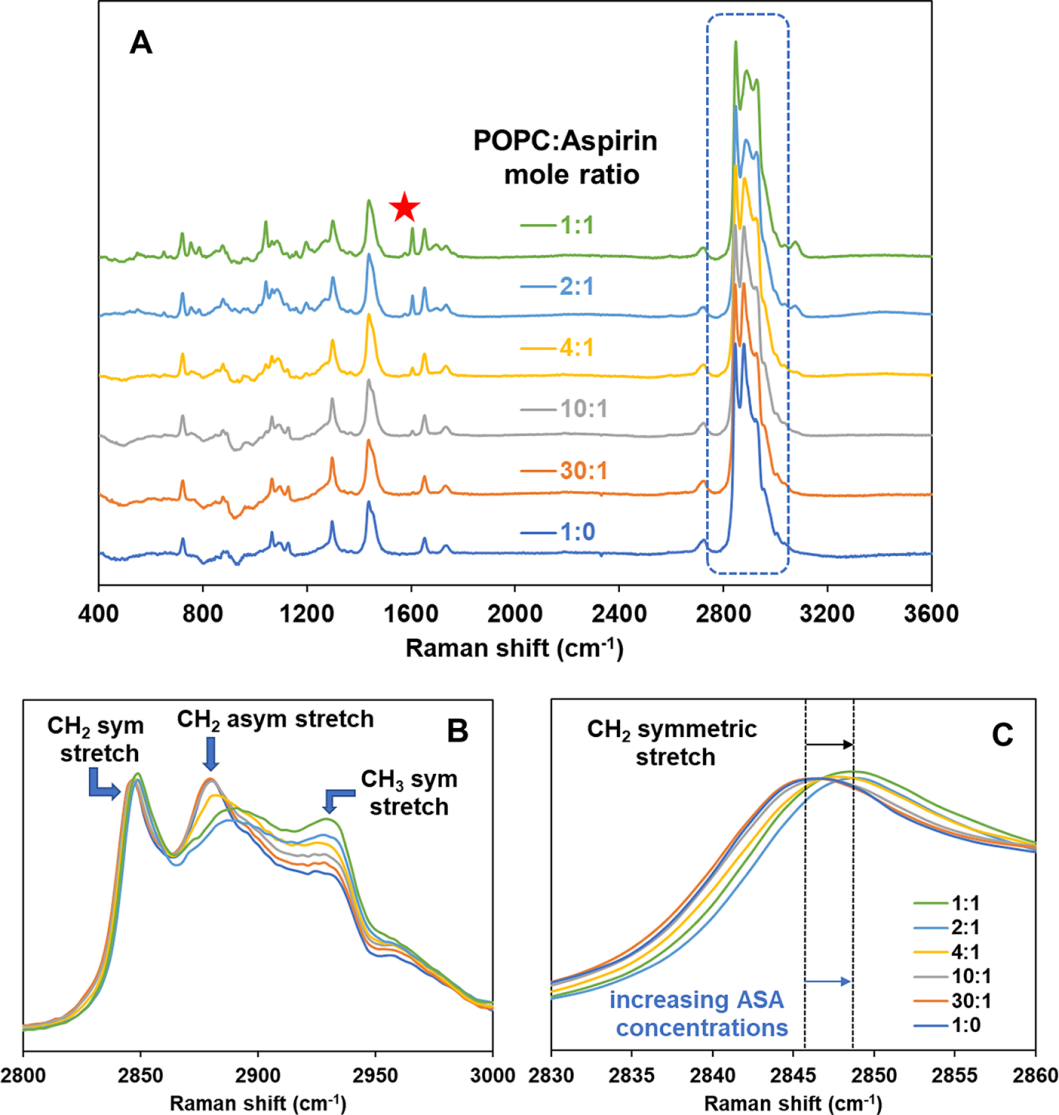
(A) Raman spectra of POPC:aspirin (mol:mol) mixtures of varying ASA concentration at pH 3 and at ambient temperature. A red star symbol indicates the most intense characteristic ASA peak at 1606 cm^–1^, (B) the Raman shift region of C–H stretching between 2800 and 3000 cm^–1^, and (C) the Raman shift region of CH_2_ symmetric stretching.

Raman microspectroscopy studies of solid-supported bilayers were performed to investigate the changes in the structural and packing properties of POPC and POPC-cholesterol bilayers upon interaction with ASA. Figure 4A shows Raman spectra at room temperature of POPC supported bilayers in the presence of varying amounts of ASA. All spectra are normalized to the intensity at 2849 cm^–1^, the most intense phospholipid peak, to allow for relative comparison. The characteristic vibration bands of POPC are observed as follows: CH_2_ twist (∼1300 cm^–1^), CH_2_ bend (∼1440 cm^–1^), C=C stretching (∼1650 cm^–1^) and C–H stretching (∼2800–3100 cm^–1^). A more detailed assignment of characteristic Raman peaks for POPC is shown in the Supporting Information (Figure S4, Table S5). Upon progressive inclusion of ASA, the expected trend of increasing intensity of the peak at 1606 cm^–1^ (aromatic C=C stretching from ASA, denoted by a star in Figure 4A) is noticeable.

**Figure 5:**
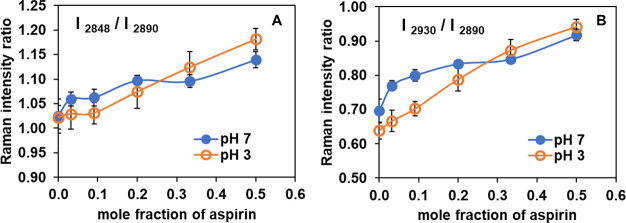
Raman intensity ratios of (A) [C–H_sym_ (2848)/C–H_asym_ (2890)], and (B) [C–H_term_ (2930)/C–H_asym_ (2890)] of POPC at ambient temperature, after subtraction of the aspirin originated peak, as a function of aspirin concentration at pH 3 (open orange circle) and pH 7 (filled blue circle). Each data point represents average and standard deviation (SD) for *n* = 5 independently prepared samples. Three different regions are scanned for each sample, and the average values are reported.

Figure 4B shows the C–H stretching region (2800 – 3000 cm^–1^), a zone that exhibits marked Raman scattering for phospholipid molecules. Its correlations to acyl chain order have been well studied.^61,62^ Peaks centered at about 2850 and 2890 cm^–1^ are assigned to the methylene C–H symmetric and C–H asymmetric stretching modes, respectively, whilst the ∼2930 cm^–1^ peak is assignable to the terminal methyl C–H symmetric stretching mode. Upon an increasing concentration of ASA in POPC, it is evident that the symmetric C–H methylene stretch band shifts to higher wavenumber, as shown with a vertical dotted line in Figure 4C. Also shown in Figure 4B is an intensity decrease of the asymmetric C–H methylene stretch band, as well as a broadening and shift to higher wavenumbers (from 2880 to 2891 cm^–1^). The frequencies of the symmetric and asymmetric C–H methylene stretch peak represent the level of conformational order and interchain coupling in the lipid chains; in general, increasing frequencies signify increasing chain decoupling.^61^ Numerous prior studies have established that ratios of relative intensities for selected peaks in the C–H stretching regions are useful indicators for determining chain decoupling, rotational disorder, relative hydrocarbon chain order/disorder parameters, and packing effects.^61−63^Figure 5 shows the ratios of peak intensity of [C–H_sym_ (2848)/C–H_asym_ (2890)] and [C–H_term_ (2930)/ C–H_asym_ (2890)]. The corresponding data from the Raman intensity ratio is shown in Supporting Information (Table S6). It is noted that the C–H stretching region (2800–3000 cm^–1^) also has peaks from ASA, that interfere with peaks from POPC. Therefore, the Raman intensity ratios shown in Figure 5 are the resultant ratios after subtraction of the aspirin-originating peaks from the spectra of the mixture of POPC and ASA. The detailed spectral subtraction method is described in the Supporting Information (Figure S5).

As seen in Figure 5, an increased concentration of ASA in POPC leads to an increase in the peak intensity ratio of [C–H_sym_ (2848)/C–H_asym_ (2890)] and [C–H_term_ (2930)/C–H_asym_ (2890)] for both pH 3 and pH 7. However, the degree of increase in each Raman intensity ratio as a function of mole fraction of ASA in POPC, is greater at pH 3 than at pH 7. The intensity ratio of [C–H_sym_ (2848)/C–H_asym_ (2890)] is considered as a signal measure of lateral packing density of hydrocarbon chains: *increasing* values of this Raman intensity ratio indicates *decreased* packing efficiency.^61^Figure 5A relates to this intensity ratio. In addition, an *increase* in the ratio of [C–H_term_ (2930)/C–H_asym_ (2890)] infers a *decrease* in both intramolecular (*gauche*/*trans*) and intermolecular (chain packing) interaction (Figure 5B). Since these two ratios both increase with increased ASA content, it thus appears that interactions of ASA molecules with the POPC lipid bilayer affect intermolecular interactions in the acyl chain region, progressively lessening packing order, thereby having a disordering effect on POPC lipid bilayers. With increased ASA concentration, the acyl chains decouple (decrease in intermolecular interactions), which would thereby increase rotational and vibrational freedom of the terminal methyl group, resulting in increased ratios of [C–H_term_ (2930)/C–H_asym_ (2890)]. All of these effects appear to be more pronounced when ASA is in a protonated, uncharged state at pH 3, compared to when it is in a charged state at pH 7. Our findings are consistent with an earlier vibrational study of the effect of NSAIDs (including ASA) on soy PC bilayer under conditions of different pH, using FTIR.^18^ This latter study revealed large changes in the vibrational peaks of PC near the aspirin's pK_a_ of 3.5, while no significant changes were seen at neutral pH, suggesting that ASA interacts more strongly with soy PC when ASA is in its neutral form. Our Raman data are consistent with a capability for ASA to interact with the acyl chain of POPC and interrupt lipid packing, increasing membrane disorder and fluidizing the lipid bilayer, with greater extent of interaction at low pH than at pH 7.

**Figure 6:**
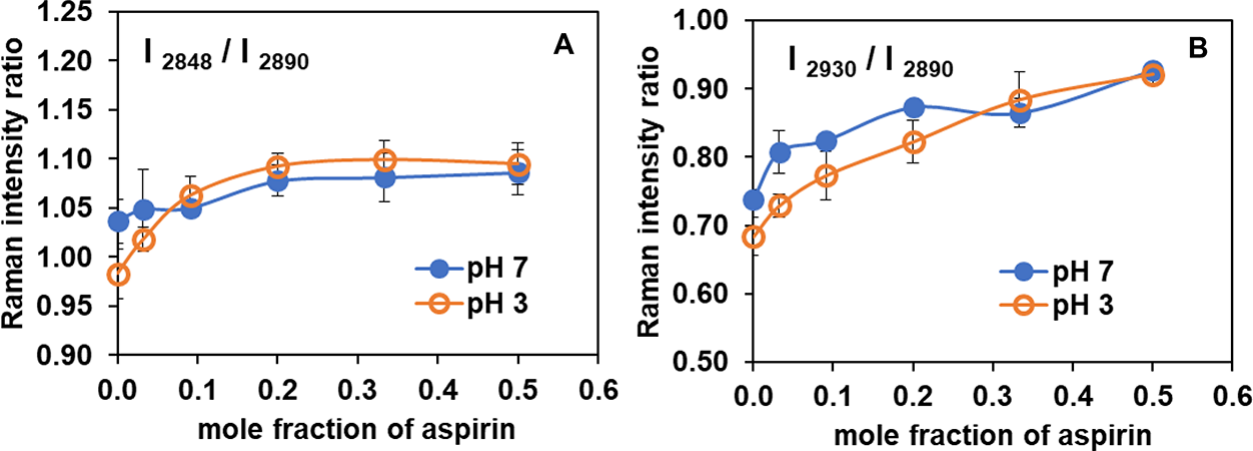
Raman intensity ratios of (A) [C–H_sym_ (2848)/C–H_asym_ (2890)], and (B) [C–H_term_ (2930)/C–H_asym_ (2890)] of POPC:chol (10:1 mole ratio) at ambient temperature (after subtraction of ASA originated peak), as a function of ASA concentration, at pH 3 (open orange circle) and pH 7 (filled blue circle). Each data point represents average and standard deviation (SD) for *n* = 5 independently prepared samples. Three different regions are scanned for each sample, and the average values are reported.

Figure 6 shows the analogous structure-rich Raman intensity ratios for selected vibrations in the C–H stretching region for POPC:chol at 10:1 mol ratio with increasing ASA concentrations. The corresponding data from Raman intensity ratio is shown in Supporting Information (Table S6). At higher concentrations of cholesterol (viz., 4:1 and 1:1 mol ratio of POPC:chol), the Raman bands around the C–H stretching region (2800 – 3100 cm^–1^) are too complex to analyze due to the presence of overlapping peaks from cholesterol. Hence the Raman analysis of POPC:chol mixtures are limited to low concentrations of cholesterol (10:1 mole ratio of POPC:chol).

A trend of increasing peak intensity ratio for both [C–H_sym_ (2848)/C–H_asym_ (2890)] and [C–H_term_ (2930)/C–H_asym_ (2890)] has been found with increased concentration of ASA, at both pH 3 and pH 7, as seen in Figure 6. As in the case of chol-free POPC, the degree of increase is greater at pH 3 than at pH 7. However, when compared to POPC without cholesterol, the effect of ASA on peak intensity ratio is less for both [C–H_sym_ (2848)/C–H_asym_ (2890)] and [C–H_term_ (2930)/C–H_asym_ (2890)], indicating that while aspirin molecules affect intermolecular interactions in the acyl chain region of mixed bilayer of POPC:chol (10:1 mole ratio), its interaction is relatively weaker, leading to less perturbation of the acyl chain packing and disordering effect compared to that in the absence of cholesterol. This is consistent with our findings in which the relative percentage change in osmotic water permeability is greater for POPC compared to that of POPC: chol (10:1 mol ratio) and that the extent of changes is greater at pH 3 than at pH 7, providing additional evidence that the effect of ASA depends on the charge state and composition of the membrane (cholesterol concentrations). A similar qualitative trend is observed for analogous studies for DOPC and DOPC with chol (10:1 mole ratio) at pH 3 (Figures S6, S7, and Table S7 in Supporting Information).

### Interfacial Properties

Interfacial tensiometry of the bilayer was used to investigate the interaction of ASA with membranes composed of DOPC and DOPC:chol at 1:1 mole ratio. Changes in bilayer tension (γ_B_) upon adsorption of ASA molecules can be gauged using the DIB system. The bilayer tension metric is related to the rigidity and stability of a biomembrane,^64^ and has been associated with the activity of cells in membrane fusion and protein function.^65^ In the droplet interface bilayer system, there is always a positive, measurable bilayer tension owing to the geometry of the oil-lipid-water system; note that the non-zero bilayer tension is due to the topology of the DIB and is not due to the presence of oil solvent within the bilayer since even solventless planar bilayers have positive tension.^66,67^ Perturbations of bilayer tension can be a result of membrane interaction. In the DIB system, bilayer tension γ_B_ can be readily derived from monolayer interfacial tension (γ_m_) at the oil-lipid-water interface, and the contact angle (*θ*) between two adherent droplets, in accordance with equation 2.
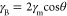
2

In addition, the free energy of formation of DIB provides the driving force for the spontaneous formation of a lipid bilayer at the interface when apposing monolayers adhere, and can be obtained by following the Young–Dupré equation 3.^68,69^
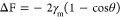
3

Effects of varying concentrations of ASA at pH 3 on bilayer tension (γ_B_) and energy of formation ΔF of DOPC membranes, are shown in Table 2. The interfacial tension of the DOPC monolayer (γ_m_) decreased from 1.12 to 0.92 with an increased mole fraction of ASA (from 0 to 0.50). Table 2 also shows that an increase of ASA concentration in the aqueous phase engenders a reduction in bilayer tension, from 1.80 mN/m in the absence of ASA, to 1.66 mN/m in the presence of 0.50 mole fraction of ASA. The reduction in bilayer tension was accompanied by a decrease in absolute value of the free energy of formation, from 0.445 mJ/m^2^ to 0.185 mJ/m^2^ at an ASA mole fraction of 0.50. There is a plausible association between changes in free energy of formation and passive transport properties for small molecules across a bilayer. This is evidenced by a recent report studying passive transport of small fluorophores across the DIB, in which the relative membrane lateral pressure (π = γ_m_(1 – cosθ), values for which are numerically half the free energy of formation) was used to demonstrate a relationship with the permeability: the weaker the relative lateral pressure (and thus free energy of formation), the greater the permeability.^70^ Our results are qualitatively consistent with the latter, namely, that increased water permeability scaled with a decreased energy of formation. The reliability of the values for interfacial tension and contact angle (and thus bilayer tension and free energy of formation) should not be significantly perturbed by any inclusion of squalene oil solvent from the surroundings of the respective interfaces. It has been observed that bilayers formed from phospholipids dispersed in squalene exhibit very high specific capacitance values, indicative of a thin “solvent-free” bilayer.^71−73^ That is because such a large hydrophobic molecule will experience a high entropic penalty in its interactions with lipid acyl chains and tend to be largely excluded from the bilayer. Any residual squalene in the bilayer would be most probably oriented parallel to the membrane plane (i.e., between its two leaflets), as seen by neutron diffraction measurements, and not situated at the water-lipid interface.^74^

**Table 2:** Interfacial Parameters for the Water/DOPC/SqE and Water/DOPC:chol/SqE Interfaces in the Presence of ASA at pH 3 and 25 °C

DOPC to ASA mole ratio	monolayer tension, γ_m_ (mN/m)^a^	contact angle, *θ* (degrees)^b^	bilayer tension, γ_B_ = 2γ_m_cosθ (mN/m)	|free energy of formation| (mJ/m^2^)
1:0	1.12 ± 0.06	36.7 ± 0.4	1.80	0.445
10:1	1.07 ± 0.06	35.2 ± 0.4	1.74	0.391
4:1	1.02 ± 0.07	33.1 ± 0.3	1.71	0.331
2:1	0.92 ± 0.06	27.1 ± 0.5	1.64	0.203
1:1	0.92 ± 0.05	25.9 ± 0.6	1.66	0.185

DOPC:chol (1:1) to ASA mole ratio	monolayer tension, γ_m_ (mN/m)^a^	contact angle, *θ* (degrees)^b^	bilayer tension, γ_B_=2γ_m_cosθ (mN/m)	|free energy of formation| (mJ/m^2^)
1:0	1.12 ± 0.09	36.4 ± 0.6	1.81	0.437
10:1	1.00 ± 0.06	35.2 ± 0.5	1.64	0.366
4:1	1.07 ± 0.08	32.1 ± 0.3	1.81	0.327
2:1	0.96 ± 0.05	33.1 ± 0.5	1.62	0.313
1:1	0.92 ± 0.04	28.7 ± 0.4	1.61	0.224

a Each data represents the average for at least 10 independent samples.

b Each data represents the average for at least 20 independent samples.

Also shown in Table 2 are the interfacial parameters for cholesterol-enriched membranes (DOPC:chol at 1:1 mole ratio) in the presence of ASA, which can be compared to the analogous case of cholesterol-free DOPC. Similar effects are seen upon inclusion of ASA as with chol-free bilayers, but with a somewhat diminished intensity. There was seen a reduction in the free energy of formation in the presence of cholesterol from 0.437 to 0.224 mJ/m^2^, as compared to the change from 0.445 to 0.185 mJ/m^2^ in the absence of cholesterol. Our data showing a relative insensitivity in energy of bilayer formation to the presence of ASA molecules for cholesterol-rich membranes, is in line with our observation of relatively modest increase in water permeability as well as less disorder in the acyl chain packing parameters, as seen in the Raman spectroscopic studies for the cholesterol-containing system. A truncated data set for the interfacial parameters collected at pH 7 is also shown in Table S8 of the Supporting Information. As with the data at pH 3, a reduction in the free energy of formation is seen with increasing incorporation of ASA, for both cholesterol-free and cholesterol-containing DOPC bilayers.

## Conclusions

Upon interaction, exogenous drugs modulate the structural and physical properties of membranes and may affect the conformation of inserted proteins, perturbing the membrane-hosted biological functions. To ascertain the effect of ASA, a classic NSAID drug, on the physicochemical properties of a membrane, we undertook systematic investigation of the interaction of ASA with a model lipid bilayer of varying cholesterol concentration. Our studies included: investigation of water permeability across DIB membranes to deduce dynamical membrane properties; thermotropic properties by DSC; structural properties by confocal Raman microspectroscopy; and interfacial tensiometry. The combined results from these diverse experimental techniques provide evidence for non-specific interaction between ASA and mixed membranes of POPC (or DOPC) with cholesterol. Our findings—of increased water permeability, lowered *T*_m_ with decreased Δ*H*, increased disorder/decreased packing efficiency, and reduced bilayer tension—indicate that ASA molecules interact with lipid bilayer in a way to weaken intermolecular forces of acyl chains and thereby enhance chain decoupling, making the overall bilayer more fluidic for both cholesterol-free and cholesterol-enriched model membranes (POPC or DOPC). However, the extent of interaction depends strongly on the concentration of cholesterol in the lipid bilayer as well as the concentrations and the charge state of ASA molecules: a greater increase in water permeability and disorder is seen when ASA is in the uncharged state. Given a molecular structure that includes three polar oxygen atoms, the interactions of ASA with the membrane are likely electrostatic in origin, partitioning into the headgroup of the lipid bilayer when ASA is in the charged state. However, in its uncharged state, ASA may penetrate deeper into the acyl chain and is capable of perturbing the order of the lipid bilayer even in the presence of chol, with potential competition in the same acyl chain region. These results are consistent with prior MD simulations demonstrating that the charge state of ASA provokes large differences in free energy profiles for its partitioning in DPPC lipid bilayers: free energies of partition (−51.5 kJ/mol) for ASA neutral version compared to its corresponding charged form (−16.9 kJ/mol).^19^ The displayed ability of ASA molecules to enhance bilayer water permeability in the presence of cholesterol is indicative of a refluidizing effect of ASA (in neutral state) on relatively more condensed cholesterol containing lipid membranes. This is further corroborated by both data from DSC showing a reduction of the cooperativity of the main phase transitions and by increasing acyl chain disorder from Raman studies. The nature and extent of interaction between ASA and the lipid bilayer is influenced by various factors, and result in aspirin having a capability for fluidizing the lipid membrane in both cholesterol-free and cholesterol-rich state, especially when uncharged, thereby counteracting the condensing effect of cholesterol.^21^ Overall, our findings add a wealth of experimental evidence for aspirin’s ability to induce bilayer disorder in the cholesterol-free fluid state and cholesterol-enriched condensed state, which may have significant consequences for its potential beneficial effects as well as its toxicity associated with gastrointestinal damage.

## Supporting Information Available

Water permeability analysis using model membrane formed by the DIB method; Contact angle measurement; Water permeability coefficients for POPC and DOPC; DSC data at pH 3 and pH 7 for DOPC and mixed DOPC:Chol MLVs; Raman spectra of pure POPC supported lipid bilayer and ASA along with the characteristic POPC peak assignments; Raman intensity ratios of POPC, POPC:chol; Raman spectra and Raman intensity ratios of DOPC and DOPC:chol with ASA; the Raman spectral subtraction method allowing for the elimination of the ASA components, and interfacial parameters for the water/DOPC/SqE and water/DOPC:chol/SqE interfaces in the presence of ASA at pH 7 are provided (PDF)
