# Supplementary material for: Aspirin Interacts with Cholesterol-Containing Membranes in a pH-Dependent Manner
Source: Langmuir. 2023 Nov 8;39(46):16444–56. doi: 10.1021/acs.langmuir.3c02242 (PMC10666536; doi:10.1021/acs.langmuir.3c02242)
Supplement: Supplementary file 1 — la3c02242_si_001.pdf [file la3c02242_si_001.pdf]

*Supporting Information*

# Aspirin Interacts with Cholesterol-Containing Membranes in a pH-Dependent Manner

*Michael Krmic, Escarlin Perez, Patrick Scollan, Katherine Ivanchenko, Alondra Gamez Hernandez, Joseph Giancaspro, Juan Rosario, Jasmin Ceja-Vega, Jamie Gudyka, Riley Porteus, Sunghee Lee**

Department of Chemistry and Biochemistry, Iona University, 715 North Avenue, New Rochelle, New York 10801, USA

*To whom correspondence should be addressed. Tel: 914-633-2638. Fax: 914-633-2240.
E-mail: SLee@iona.edu

**List of Supplemental Figures**

**Figure S1.** Schematics of (A) aqueous microdroplets surrounded by self-assembled structures, for use as a biomembrane model, and (B) typical DIB-based osmotic water permeability measurement

**Figure S2.** A microscopic picture of two adherent droplets in SqE in the presence of DOPC 5 mM. $R_1$ and $R_2$ are the radii of the respective two droplets and r is the radius of the contact zone between the droplets, and the contact angle (θ) is determined by the eqn. 3. The scale bar on the image represents 100 μm.

**Figure S3.** The relative percentage change (%) in osmotic water permeability ($P_f/P_f^o$, where $P_f^o$ represents the osmotic water permeability in the absence of ASA) of DOPC and mixed bilayer formed from DOPC:chol (1:1 mole ratio) at 30°C with varying mole fraction of ASA, at (A) pH 3 and (B) pH 7.

**Figure S4.** Raman spectra of pure POPC lipid bilayer (film of POPC liposome) and aspirin (dried film) at room temperature.

**Figure S5.** (A) Raman spectra of POPC and aspirin mixture (POPC to aspirin of 1 to 1 mole ratio at pH 3) in the C–H stretching region, the aspirin spectra are scaled to the intensity of the 1606 $cm^{-1}$, the solid green line shows the spectrum of POPC after subtraction of the aspirin spectrum (solid orange line) from the original spectrum (dotted green line), (B) superposition of

spectra of POPC and aspirin lipid mixtures in the Raman shift region between 2800 and 3000 $cm^{-1}$ after subtraction.

**Figure S6.** (A) Raman spectra of DOPC:aspirin (mol:mol) mixtures of varying ASA concentration at pH 3 and at ambient temperature, (B) Raman shift region of C−H stretching between 2800 and 3000 $cm^{-1}$ (comparison between DOPC vs DOPC:aspirin (3:1 mole ratio).

**Figure S7.** Raman intensity ratios of [C−$H_{sym}$ (2848)/C−$H_{asym}$ (2890)] and [C−$H_{term}$ (2930)/C−$H_{asym}$ (2890)] of (A) DOPC and (B) DOPC:chol (10:1 mole ratio) at ambient temperature, (after subtraction of ASA originated peak) at pH 3. Corresponding Raman intensity ratios are given in Table S6.

**List of Supplemental Tables**

**Table S1.** Effect of aspirin on osmotic water permeability (μm/s) at 30°C for POPC and mixed POPC:chol bilayer at pH 3 and pH 7.

**Table S2.** Effect of aspirin on osmotic water permeability (μm/s) at 30°C for DOPC and mixed DOPC:chol bilayer at pH 3 and pH 7.

**Table S3.** Thermodynamic parameters ($T_m$ and ΔH) for main phase transition of DOPC MLVs at different concentration of aspirin at pH 3 and pH 7.

**Table S4.** Thermodynamic parameters ($T_m$ and ΔH) for main phase transition of mixed DOPC:chol MLVs at different concentration of aspirin at pH 3 and pH 7.

**Table S5.** Peak assignments of POPC Raman spectra

**Table S6.** Raman intensity ratios of [C−$H_{sym}$ (2848)/C−$H_{asym}$ (2890)] and [C−$H_{term}$ (2930)/C−$H_{asym}$ (2890)] of POPC and POPC:chol (10:1 mole ratio) at ambient temperature. The corresponding plots are shown in the main article (Figure 4 for POPC and Figure 5 for POPC:chol).

**Table S7.** Raman intensity ratios of [C−$H_{sym}$ (2848)/C−$H_{asym}$ (2890)] and [C−$H_{term}$ (2930)/C−$H_{asym}$ (2890)] of DOPC and DOPC:chol (10:1 mole ratio) at ambient temperature.

**Table S8.** Interfacial parameters for the water/DOPC/SqE and water/DOPC:chol/SqE interfaces in the presence of ASA at pH 7, and 25 °C

## 1. Water permeability analysis using model membrane formed by the droplet interface bilayer (DIB) method

The water permeability measurement was performed using the model membrane formed by the droplet interface bilayer (DIB) method. A DIB is formed when aqueous microdroplets bounded by lipid monolayers create a region that has a structure essentially the same as the double-leaflet lipid bilayer of cell membranes (Figure S1A).

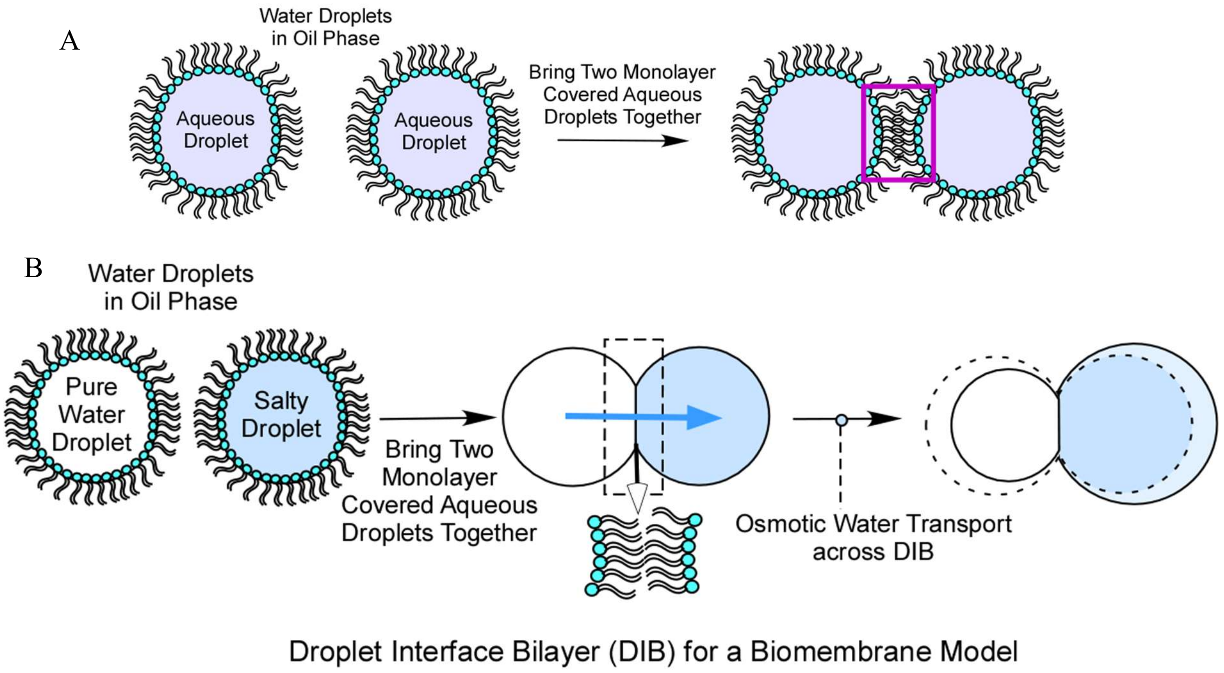


**Figure S1.** Schematics of (A) aqueous microdroplets surrounded by self-assembled structures, for use as a biomembrane model, and (B) typical DIB-based osmotic water permeability measurement (Adaped from Lee, Sunghee. "Good to the last drop: interfacial droplet chemistry, from crystals to biological membranes." Accounts of chemical research 51, no. 10 (2018): 2524-2534., Copyright 2018 American Chemical Society.)

When two osmotically unbalanced microdroplets were made to adhere at a bilayer, the osmotic gradient drives water transport through the droplet bilayer (the direction of water transport is shown with the arrow in Figure S1B), resulting in a visible change in droplet diameter. Any electrolyte flux is expected to be negligible compared to that of water, as ion permeation is typically almost eight orders of magnitude slower than that of water. The corresponding changes in droplet volume over time (dV/dt) is measured optically by microscopic observation; and the behavior of the system follows the expression of equation (1) based on Fick's Law:

$$\frac{dV(t)}{dt} = -P_f A(t) v_w \Delta C(t) \quad (1)$$

where A is the geometric bilayer area, $\nu_w$ is the molar volume of water (18 mL/mol), **Δ**C(t) is the osmolality gradient between two droplets, and $P_f$ is the bilayer permeability coefficient of water. The volume change with time (dV/dt) is related to the bilayer permeability coefficient of water, $P_f$, as expressed in the Equation (1). When the bilayer contact area is constant, the time evolution of the swelling droplet can be obtained from the following equation derived from the integration of eqn. 1, with the following simplifying assumption: since one of the droplets (the shrinking droplet) contains no osmotic agent, its concentration does not change with time:[1, 2]

$$\left(\frac{V}{V_o}\right)^2 = \left(\frac{2P_f A v_w C_o}{V_o}\right)t + 1 \quad (2)$$

Using the measured values for: initial size of the osmotic (swelling) droplet; bilayer contact area (A); and initial osmolarity of the osmotic droplet ($C_o$), then the coefficient $P_f$ for bilayer water permeability may be derived from eqn. 2 from the slope of the curve obtained by plotting $(V/V_o)^2$ as a function of time. All data points presented in this paper are an average ($n \geq 30$) of individual permeability runs, each of which took place over a time course (~5 min) for osmotic water movement across the droplet bilayer, during which time the droplet contact area (A) remains constant. The recorded videos and images were post-analyzed to measure the dimension of droplets and contact area using custom built image analysis software. All droplet pairs had substantially the same initial size relative to each other, in the diameter range of 100 ± 5 μm diameter.

## 2. Contact Angle Measurement

For the contact angle (θ) measurement, two apposing iso-osmotic droplets are made to contact with each other. From the microscopic video images of the two adherent droplets, the contact angle can be measured by considering the geometry of the contacting spheres (as given in eqn. 3) based on geometrical parameters shown in Figure S2,

$$2\theta = \sin^{-1}\left(\frac{r}{R_1}\right) + \sin^{-1}\left(\frac{r}{R_2}\right) \quad (3)$$

where, $R_1$ and $R_2$ are the radii of the respective two droplets and r is the radius of the contact zone between the droplets.[49-50] Reported values are from the average of 10 or more measurements.

[1] Lopez, M.; Evangelista, S. E.; Morales, M.; Lee, S. Enthalpic effects of chain length and unsaturation on water permeability across droplet bilayers of homologous monoglycerides. *Langmuir* 2017, 33 (4), 900-912.
[2] Thiam, A. R.; Bremond, N.; Bibette, J. From stability to permeability of adhesive emulsion bilayers. *Langmuir* 2012, 28 (15), 6291-6298.

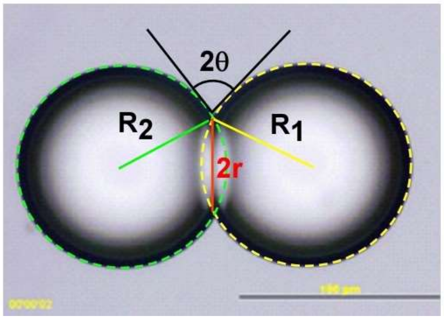


**Figure S2.** A microscopic picture of two adherent droplets in SqE in the presence of DOPC 5 mM. $R_1$ and $R_2$ are the radii of the respective two droplets and r is the radius of the contact zone between the droplets, and the contact angle (θ) is determined by the eqn. 3. The scale bar on the image represents 100 μm. (Adapted from Wood, Megan, Michael Morales, Elizabeth Miller, Samuel Braziel, Joseph Giancaspro, Patrick Scollan, Juan Rosario, Alyssa Gayapa, Michael Krmic, and Sunghee Lee. "Ibuprofen and the phosphatidylcholine bilayer: membrane water permeability in the presence and absence of cholesterol." Langmuir 37, no. 15 (2021): 4468-4480. Copyright 2021 American Chemical Society)

## 3. Water permeability coefficients

The osmotic water permeability coefficients ($P_f$) of mixed bilayers of POPC:chol at 30°C as a function of varying mole fraction of aspirin, at both pH 3 and pH 7 are shown in Table S1.

**Table S1.** Effect of aspirin on osmotic water permeability (μm/s) at 30°C for POPC and mixed POPC:chol bilayer at pH 3 and pH 7.*

| POPC or POPC:chol to Aspirin mole ratio | POPC, μm/s | | POPC:chol (10:1 mole), μm/s | | POPC:chol (4:1 mole), μm/s | | POPC:chol (1:1 mole), μm/s | |
|---|---|---|---|---|---|---|---|---|
| | pH 3 | pH 7 | pH 3 | pH 7 | pH 3 | pH 7 | pH 3 | pH 7 |
| 1 : 0 | 71 ± 4 | 77 ± 3 | 68 ± 3 | 73 ± 3 | 65 ± 3 | 70 ± 3 | 62 ± 3 | 67 ± 3 |
| 30 : 1 | 76 ± 3 | 80 ± 4 | 73 ± 3 | 74 ± 5 | 67 ± 4 | 70 ± 5 | 63 ± 3 | 67 ± 5 |
| 10 : 1 | 80 ± 5 | 83 ± 4 | 75 ± 4 | 75 ± 3 | 70 ± 4 | 70 ± 4 | 65 ± 4 | 68 ± 5 |
| 4 : 1 | 84 ± 4 | 84 ± 3 | 76 ± 4 | 75 ± 4 | 70 ± 3 | 70 ± 4 | 66 ± 5 | 67 ± 4 |
| 1 : 1 | 88 ± 5 | 85 ± 3 | 77 ± 5 | 75 ± 3 | 70 ± 5 | 70 ± 3 | 63 ± 5 | 66 ± 5 |

* Each data represents an average of individual permeability runs (n>30 independent samples) and standard deviation as error bars.

**Table S2.** Effect of aspirin on osmotic water permeability (μm/s) at 30°C for DOPC and mixed DOPC:chol bilayer at pH 3 and pH 7.

| DOPC or DOPC:chol to Aspirin mole ratio | DOPC, μm/s | | DOPC:chol (1:1 mole), μm/s | |
|---|---|---|---|---|
| | pH 3 | pH 7 | pH 3 | pH 7 |
| 1 : 0 | 74 ± 4 | 78 ± 3 | 67 ± 3 | 71 ± 3 |
| 10 : 1 | 83 ± 5 | 83 ± 5 | 68 ± 4 | 70 ± 4 |
| 4 : 1 | 87 ± 3 | 85 ± 5 | 69 ± 3 | 72 ± 4 |
| 2 : 1 | 87 ± 3 | 85 ± 3 | 69 ± 3 | 70 ± 5 |
| 1 : 1 | 90 ± 6 | 87 ± 3 | 68 ± 5 | 72 ± 5 |

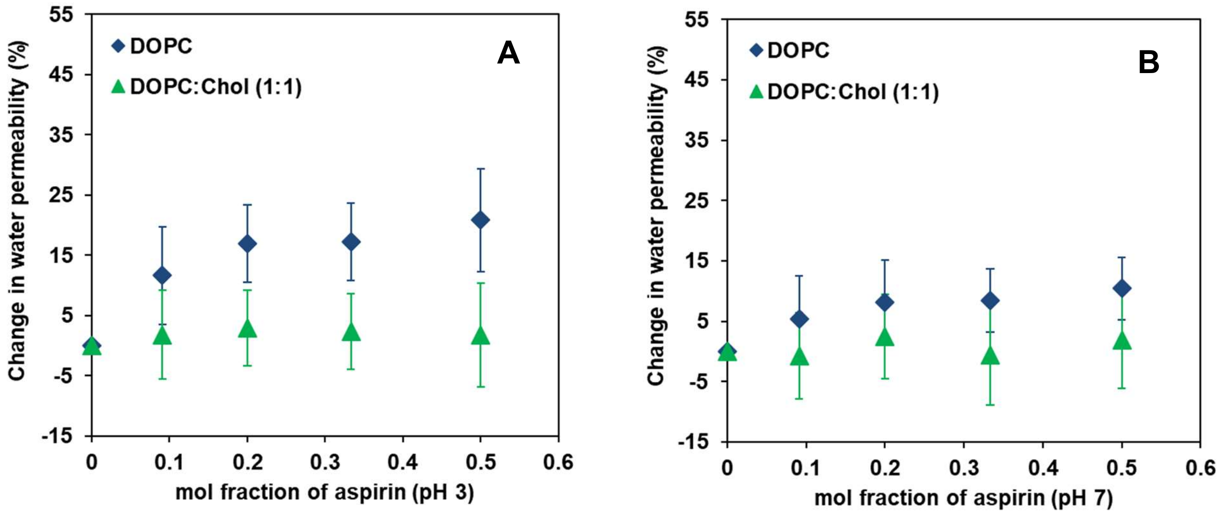


**Figure S3.** The relative percentage change (%) in osmotic water permeability ($P_f/P_f^o$, where $P_f^o$ represents the osmotic water permeability in the absence of ASA) of DOPC and mixed bilayer formed from DOPC:chol (1:1 mole ratio) at 30°C with varying mole fraction of ASA, at (A) pH 3 and (B) pH 7.

## 4. Thermotropic property

The thermodynamic data, $T_m$ and ΔH, from endothermic DSC thermograms for mixed DOPC:chol MLVs in the presence of different concentrations of aspirin at pH 3 are shown in Table S2 and S3.

**Table S3.** Thermodynamic parameters ($T_m$ and ΔH) for main phase transition of DOPC MLVs at different concentration of aspirin at pH 3 and pH 7.

| DOPC:Aspirin (mol:mol) | DOPC (at pH 3) | | DOPC (at pH 7) | |
|---|---|---|---|---|
| | $T_m$ (°C) | ΔH (kcal/mol) | $T_m$ (°C) | ΔH (kcal/mol) |
| 1:0 | −17.58 ± 0.10 | 9.59 ± 0.35 | −17.86 ± 0.12 | 9.26 ± 0.37 |
| 30:1 | −18.09 ± 0.02 | 7.67 ± 0.50 | −18.15 ± 0.06 | 7.76 ± 0.33 |
| 15:1 | −18.58 ± 0.20 | 6.06 ± 0.40 | −18.90 ± 1.06 | 6.21 ± 0.21 |
| 7:1 | −19.83 ± 0.43 | 4.00 ± 0.15 | −19.70 ± 1.31 | 5.11 ± 0.36 |
| 3:1 | −23.23 ± 0.52 | 1.33 ± 0.25 | −22.00 ± 2.01 | 1.81 ± 0.40 |

**Table S4.** Thermodynamic parameters ($T_m$ and ΔH) for main phase transition of mixed DOPC:chol MLVs at different concentration of aspirin at pH 3 and pH 7.

| Lipid (DOPC:chol): Aspirin (mol:mol) | DOPC:chol (10:1) | | | |
|---|---|---|---|---|
| | pH 3 | | pH 7 | |
| | $T_m$ (°C) | ΔH (kcal/mol) | $T_m$ (°C) | ΔH (kcal/mol) |
| 1:0 | −18.05 ± 0.03 | 6.14 ± 0.50 | −18.44 ± 0.30 | 5.83 ± 0.65 |
| 30: 1 | −18.29 ± 0.05 | 3.85 ± 0.21 | −18.83 ± 0.20 | 4.03 ± 0.19 |
| 15 : 1 | −18.76 ± 0.38 | 3.41 ± 0.34 | −19.52 ± 0.73 | 2.86 ± 0.26 |
| 7 : 1 | −19.77 ± 0.20 | 1.60 ± 0.30 | −20.98 ± 0.58 | 2.75 ± 0.35 |
| 3 : 1 | −25.84 ± 0.60 | 0.09 ± 0.05 | −26.13 ± 0.50 | 0.09 ± 0.05 |

| Lipid (DOPC:chol): Aspirin (mol:mol) | DOPC:chol (4:1) | | | |
|---|---|---|---|---|
| | pH 3 | | pH 7 | |
| | $T_m$ (°C) | ΔH (kcal/mol) | $T_m$ (°C) | ΔH (kcal/mol) |
| 1:0 | −19.36 ± 0.13 | 3.18 ± 0.31 | −20.32 ± 0.25 | 2.73 ± 0.28 |
| 30: 1 | −20.12 ± 0.02 | 2.40 ± 0.22 | −19.82 ± 0.25 | 2.18 ± 0.50 |
| 15 : 1 | −20.92 ± 0.11 | 1.79 ± 0.24 | −20.52 ± 0.10 | 2.06 ± 0.26 |
| 7 : 1 | −22.92 ± 0.16 | 0.78 ± 0.06 | −23.23 ± 0.20 | 0.80 ± 0.12 |
| 3 : 1 | NA | NA | NA | NA |

## 5. Structural property

Figure S4 shows the Raman spectra of POPC lipid bilayer (top) and aspirin (bottom) at room temperature. The detailed characteristic peak assignments for POPC are shown in Table S4.

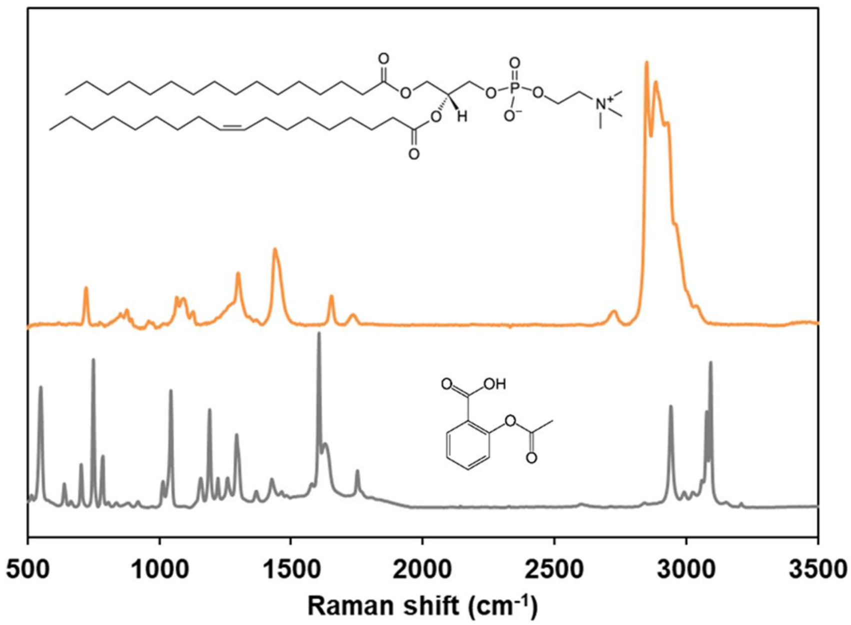


**Figure S4.** Raman spectra of pure POPC lipid bilayer (film of POPC liposome) and aspirin (dried film) at room temperature.

**Table S5.** Peak assignments of POPC Raman spectra[3]

| Raman peak ($cm^{-1}$) | Vibrational assignment |
|---|---|
| 723 | N-$CH_3$ symmetric stretch |
| 872 | N-$CH_3$ asymmetric stretch |
| 1066 | C–C trans stretch |
| 1091 | C–C gauche stretch |
| 1296 | $CH_2$ twist |
| 1438 | $CH_2$ bend |
| 1652 | C=C stretch |
| 1735 | C=O stretch |
| 2847 | $CH_2$ symmetric stretch |

[3] Giancaspro, J., Scollan, P., Rosario, J., Miller, E., Braziel, S. and Lee, S., 2022. Structural determination of model phospholipid membranes by Raman spectroscopy: Laboratory experiment. Biochemistry and Molecular Biology Education, 50(2), pp.181-192.; Czamara, K., Majzner, K., Pacia, M.Z., Kochan, K., Kaczor, A. and Baranska, M., 2015. Raman spectroscopy of lipids: a review. Journal of Raman Spectroscopy, 46(1), pp.4-20.

| | |
|---|---|
| 2880 | $CH_2$ asymmetric stretch |
| 2924 | $CH_3$ symmetric stretch |
| 3006 | unsaturated C–H stretch |
| 3036 | Choline $CH_3$ asymmetric stretch |

**Table S6.** Raman intensity ratios of [C−$H_{sym}$ (2848)/C−$H_{asym}$ (2890)] and [C−$H_{term}$ (2930)/C−$H_{asym}$ (2890)] of POPC and POPC:chol (10:1 mole ratio) at ambient temperature. The corresponding plots are shown in the main article (Figure 4 for POPC and Figure 5 for POPC:chol).

| POPC:Aspirin (mol:mol) | Raman intensity ratio | | | |
|---|---|---|---|---|
| | pH 3 | | pH 7 | |
| | I=2848/2890 | I=2930/2890 | I=2848/2890 | I=2930/2890 |
| 1:0 | 1.022 ± 0.024 | 0.639 ± 0.010 | 1.025 ± 0.035 | 0.696 ± 0.049 |
| 30:1 | 1.029 ± 0.031 | 0.667 ± 0.015 | 1.060 ± 0.014 | 0.770 ± 0.010 |
| 10:1 | 1.031 ± 0.022 | 0.703 ± 0.017 | 1.062 ± 0.018 | 0.800 ± 0.034 |
| 5:1 | 1.074 ± 0.033 | 0.788 ± 0.038 | 1.097 ± 0.005 | 0.834 ± 0.020 |
| 2:1 | 1.124 ± 0.032 | 0.873 ± 0.040 | 1.096 ± 0.013 | 0.847 ± 0.022 |
| 1:1 | 1.181 ± 0.022 | 0.942 ± 0.007 | 1.140 ± 0.017 | 0.918 ± 0.014 |

| POPC/Chol (10/1):Aspirin (mol:mol) | Raman intensity ratio | | | |
|---|---|---|---|---|
| | pH 3 | | pH 7 | |
| | I=2848/2890 | I=2930/2890 | I=2848/2890 | I=2930/2890 |
| 1:0 | 0.983 ± 0.025 | 0.684 ± 0.028 | 1.036 ± 0.022 | 0.739 ± 0.014 |
| 30:1 | 1.018 ± 0.012 | 0.729 ± 0.017 | 1.049 ± 0.041 | 0.807 ± 0.031 |
| 10:1 | 1.063 ± 0.019 | 0.773 ± 0.035 | 1.050 ± 0.008 | 0.824 ± 0.010 |
| 5:1 | 1.093 ± 0.013 | 0.823 ± 0.031 | 1.078 ± 0.016 | 0.873 ± 0.002 |
| 2:1 | 1.099 ± 0.020 | 0.884 ± 0.040 | 1.081 ± 0.025 | 0.864 ± 0.021 |
| 1:1 | 1.095 ± 0.021 | 0.921 ± 0.015 | 1.086 ± 0.023 | 0.927 ± 0.010 |

The C−H stretching region (2800 – 3000 cm$^{-1}$) also has peaks from aspirin that interfere with peaks from POPC. Therefore, appropriate subtraction of aspirin peak is necessary to monitor the effect of aspirin on the packing properties of POPC hydrocarbon chains. Before spectral subtraction, the aspirin spectra were scaled to the intensity of the 1606 cm$^{-1}$ (aromatic C=C stretching from aspirin). The resulting hydrocarbon chain contribution from POPC is shown in green solid line in Figure S4A, along with before (dotted) subtraction of the scaled aspirin spectrum (solid orange). The spectra shown in Figure S4B are those obtained after the spectral subtraction, allowing for the elimination of the aspirin components for varying mole fraction of aspirin in POPC.

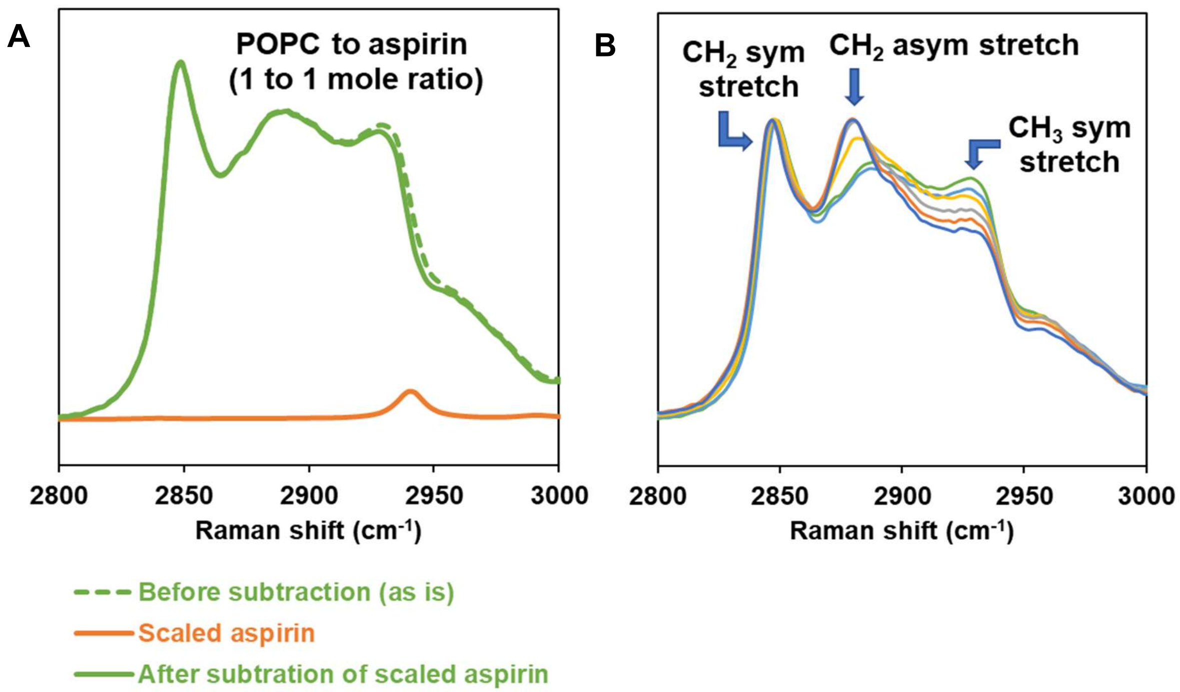


**Figure S5.** (A) Raman spectra of POPC and aspirin mixture (POPC to aspirin of 1 to 1 mole ratio at pH 3) in the C−H stretching region, the aspirin spectra are scaled to the intensity of the 1606 cm$^{-1}$, the solid green line shows the spectrum of POPC after subtraction of the aspirin spectrum (solid orange line) from the original spectrum (dotted green line), (B) superposition of spectra of POPC and aspirin lipid mixtures in the Raman shift region between 2800 and 3000 cm$^{-1}$ after subtraction.

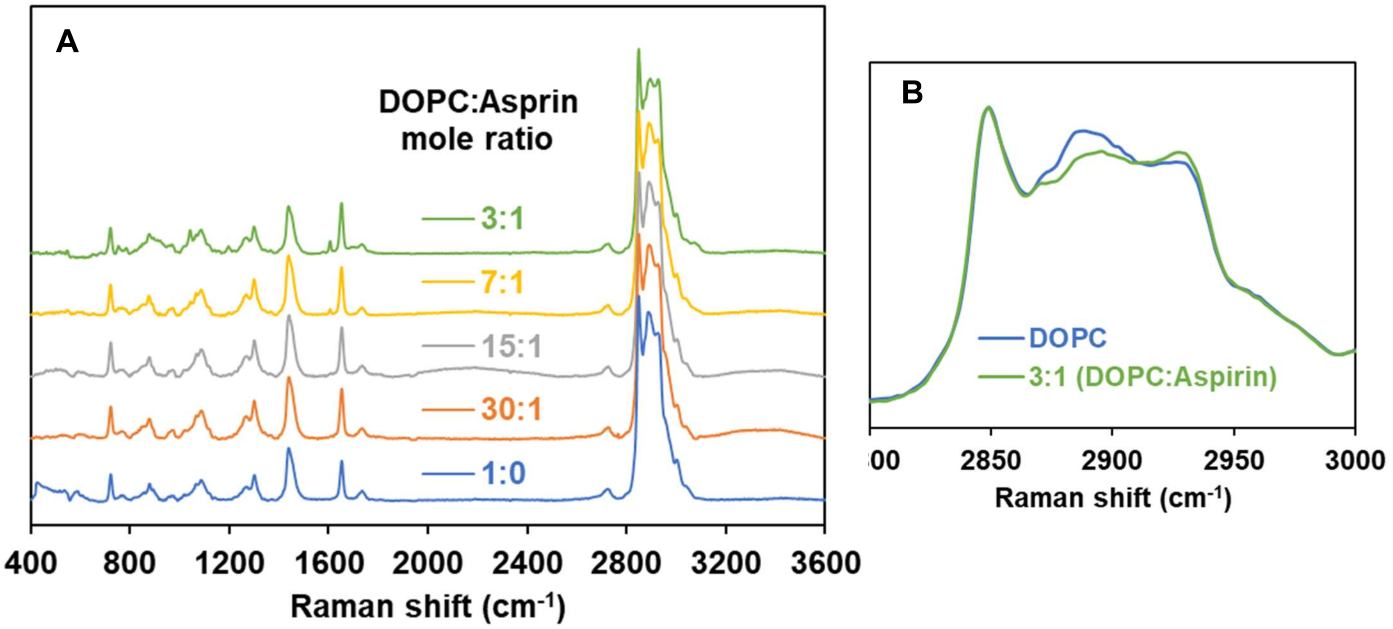


**Figure S6.** (A) Raman spectra of DOPC:aspirin (mol:mol) mixtures of varying ASA concentration at pH 3 and at ambient temperature, (B) Raman shift region of C−H stretching between 2800 and 3000 $cm^{-1}$ (comparison between DOPC vs DOPC:aspirin (3:1 mole ratio).

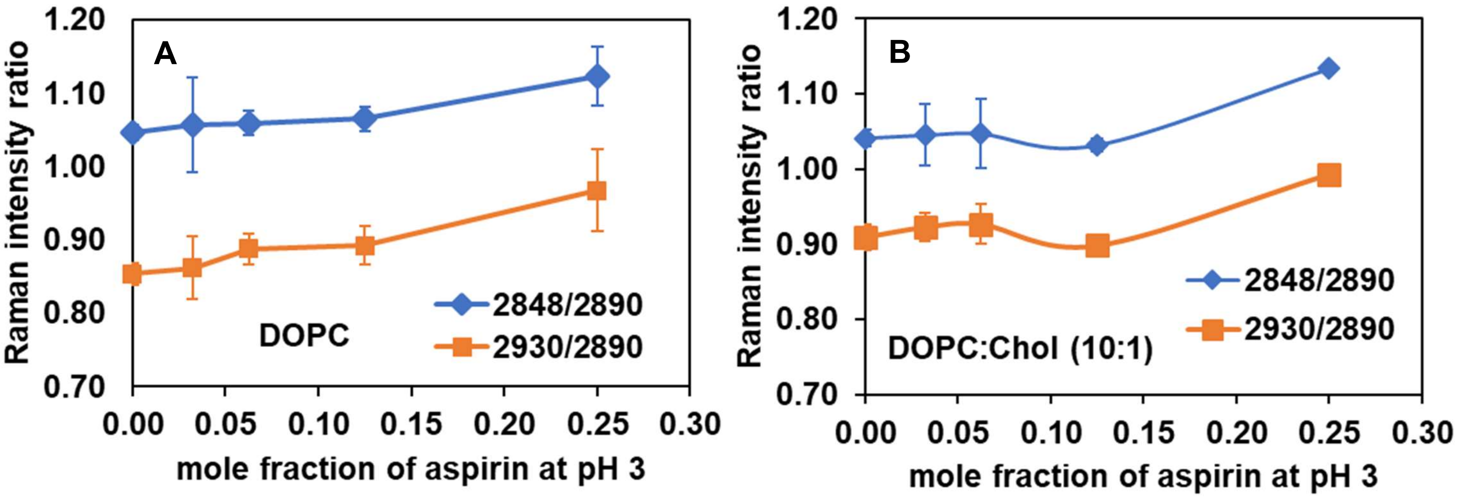


**Figure S7.** Raman intensity ratios of [C−$H_{sym}$ (2848)/C−$H_{asym}$ (2890)] and [C−$H_{term}$ (2930)/C−$H_{asym}$ (2890)] of (A) DOPC and (B) DOPC:chol (10:1 mole ratio) at ambient temperature, (after subtraction of ASA originated peak) at pH 3. Corresponding Raman intensity ratios are given in Table S7.

**Table S7.** Raman intensity ratios of [C−$H_{sym}$ (2848)/C−$H_{asym}$ (2890)] and [C−$H_{term}$ (2930)/C−$H_{asym}$ (2890)] of DOPC and DOPC:chol (10:1 mole ratio) at ambient temperature.

| lipid:Aspirin (mol:mol) | Raman intensity ratio at pH 3 | | | |
|---|---|---|---|---|
| | DOPC | | DOPC:Chol (10:1) | |
| | I=2848/2890 | I=2930/2890 | I=2848/2890 | I=2930/2890 |
| 1:0 | 1.047 ± 0.011 | 0.854 ± 0.015 | 1.041 ± 0.011 | 0.909 ± 0.017 |
| 30:1 | 1.058 ± 0.065 | 0.862 ± 0.043 | 1.045 ± 0.041 | 0.923 ± 0.019 |
| 15:1 | 1.059 ± 0.017 | 0.888 ± 0.021 | 1.047 ± 0.046 | 0.927 ± 0.026 |
| 7:1 | 1.065 ± 0.017 | 0.892 ± 0.026 | 1.032 ± 0.009 | 0.898 ± 0.008 |
| 3:1 | 1.123 ± 0.040 | 0.967 ± 0.056 | 1.134 ± 0.004 | 0.993 ± 0.005 |

## 6. Interfacial property

**Table S8.** Interfacial parameters for the water/DOPC/SqE and water/DOPC:chol/SqE interfaces in the presence of ASA at pH 7, and 25 °C

| DOPC to ASA mole ratio | monolayer tension, $\gamma_m$ (mN/m)* | contact angle, $\theta$ (degrees)** | bilayer tension, $\gamma_B=2\gamma_m\cos\theta$ (mN/m) | \|free energy of formation\| (mJ/$m^2$) |
|---|---|---|---|---|
| 10:1 | 1.11 ± 0.11 | 36.3 ± 0.5 | 1.79 | 0.431 |
| 1:1 | 1.01 ± 0.05 | 29.8 ± 0.6 | 1.75 | 0.267 |

| DOPC:chol (1:1) to ASA mole ratio | monolayer tension, $\gamma_m$ (mN/m)* | contact angle, $\theta$ (degrees)** | bilayer tension, $\gamma_B=2\gamma_m\cos\theta$ (mN/m) | \|free energy of formation\| (mJ/$m^2$) |
|---|---|---|---|---|
| 10:1 | 1.07 ± 0.05 | 36.1 ± 0.6 | 1.72 | 0.409 |
| 1:1 | 0.97 ± 0.06 | 29.5 ± 0.7 | 1.68 | 0.251 |

* Each data represents the average for 5−10 independent samples.

** Each data represents the average for 3−8 independent samples.
